# When estrogen deficiency meets immune responses induced by rabies vaccination

**DOI:** 10.1128/spectrum.02726-24

**Published:** 2025-03-25

**Authors:** Hua Qian, Junjie Zhang, Li Tian, Lele Liu, Menghua Li, Zezheng Jiang, Xiaoying Lei, Wenwen Zheng, Peilu Sun, Xuexing Zheng

**Affiliations:** 1 The Second Hospital, Cheeloo College of Medicine, Shandong University 66555, Jinan, Shandong, China; 2 Department of Virology, School of Public Health, Cheeloo College of Medicine, Shandong University 66555 , Jinan, Shandong, China; 3 Institute of Pharmacology, Shandong First Medical University 518873, Jinan, Shandong, China; Barnard College, Columbia University, New York, New York, USA

**Keywords:** rabies virus, viral neutralizing antibodies, ovariectomy, menopause, estrogen, vaccine

## Abstract

Estrogen deficiency in postmenopausal women is accompanied by immune status alterations, leading to a chronic low-grade inflammatory phenotype. Immediate rabies postexposure prophylaxis (PEP) following a transdermal bite or scratch from a rabies-infected animal is urgently needed. However, whether immune alterations in postmenopausal women influence the reaction to rabies vaccination remains unclear. Bilateral ovariectomized (OVX) and Sham mice were immunized with modified live vaccine RABV LBNSE. LBNSE immunization had no obvious pathological effect on the mice in either group and effectively protected all mice from RABV attack. Although 100% protection was found, the reduction rate of viral neutralizing antibody titers in the LBNSE-OVX mice was greater than that in the LBNSE-Sham mice. LBNSE immunization recruited/activated fewer dendritic cells (DCs) and B cells in the lymph nodes, while more B cells were detected in the blood of LBNSE-OVX mice than in that of LBNSE-Sham mice. Th1 and Th2 immune responses are both rapidly induced in LBNSE-OVX-subjected mice and are inclined toward a Th2-biased immune response. LBNSE immunization in OVX mice elicited similar amounts of RABV-specific CD4^+^ and CD8^+^ T cells as those in Sham mice. Our data revealed that the protective efficacy of rabies vaccination was slightly decreased by estrogen deficiency and that DC and B lymphocyte recruitment/activation and Th-mediated responses in splenocytes were partly altered; however, rabies vaccination offered sufficient protection against RABV within the observation period, helping alleviate anxiety related to rabies virus exposure after menopause. Additional measures might be helpful to improve long-term effective protection in postmenopausal women.

**IMPORTANCE:**

Menopause has a distinct effect on the decrease in the female immune system, and whether protection efficacy after rabies vaccination in postmenopausal women is influenced requires evaluation. Our findings demonstrated that although viral neutralizing antibody (VNA) titers in the LBNSE-OVX mice were similar to those in the LBNSE-Sham mice, VNAs declined faster than those in the LBNSE-Sham mice within the observation period. Fewer dendritic cells in the lymph nodes were recruited/activated in LBNSE-OVX mice than in LBNSE-Sham mice, whereas B cells in the lymph nodes and peripheral blood exhibited the opposite tendency. Th2-biased immune responses were induced in LBNSE-OVX mice, and no significant changes were observed in RABV-specific CD4^+^ or CD8^+^ T cells. These results provide evidence that rabies vaccination could provide effective protection for postmenopausal women within the observation period, but other measures might be needed to improve protection, which is beneficial for alleviating anxiety of menopausal women when facing rabies immunization.

## INTRODUCTION

Most vaccine clinical trials have focused on healthy people aged 18−65 years, excluding older people, pregnant women, postmenopausal women, and children (1). However, menopause is a juncture in life that every woman experiences, accounting for approximately 1/3 of a woman’s life cycle with the increasing life expectancy worldwide. Postmenopausal women exhibit a chronic low-grade inflammatory phenotype characterized by cytokine expression and immune cell alterations (2, 3). These systematic immune changes are closely associated with estrogen deficiency after menopause (4, 5). Clinical data regarding the immune phenotype of postmenopausal patients suggest that after menopause, women display increased levels of inflammatory cytokines, including interleukin 1β (IL-1β), IL-6, and tissue necrosis factor α (TNF-α) (6, 7). Moreover, immune cell numbers were shown to be altered in postmenopausal women (8). Owing to these immune alterations, postmenopausal women are more vulnerable to infectious diseases (9). Studies by animal models proposed that there could be different responses to vaccines after menopause (10).

Rabies is a viral disease caused by the rabies virus (RABV) that occurs in more than 150 countries and territories (11).Rabies causes 1.6 to 10.4 million disability-adjusted life years and 2.9–21.5 billion US dollars in economic losses globally every year (12), and approximately 35,000 people die from dog-mediated rabies in Asia every year (13), accounting for approximately 60.0% of global deaths (approximately 59,000 deaths). Once clinical symptoms occur, rabies is almost always fatal (14). There is currently no effective treatment for rabies, and prompt vaccination before or shortly after exposure is an effective strategy to combat the disease (15). The effectiveness of the rabies vaccine is determined mainly by detecting the titer of promptly produced neutralizing antibodies, and cellular immunity has also been confirmed to be involved in related immune processes. Sex differences in rabies VNA levels have been reported previously (16), indicating that gonadal hormones, including estrogen, might be involved in modulating relative immune responses. At present, it is unclear whether immune function changes resulting from estrogen deficiency in postmenopausal women affect the effectiveness of rabies vaccination.

Preventive immunization may be an overlooked and underused area in the medical concerns of midlife women. The rabies vaccine, as a therapeutic vaccine, can prevent 99% of deaths if it is administered promptly after exposure. Whether humoral immunity and cellular immunity induced by rabies vaccination are weakened in postmenopausal women needs to be clarified for clinical consultation and application. Postmenopausal women who need immediate rabies postexposure prophylaxis (PEP) following a transdermal bite or scratch from infected animals are often concerned about this. In this study, we constructed an ovariectomized (OVX) mouse model to simulate and explore the humoral and cellular immune responses of postmenopausal women after rabies vaccine inoculation. This study aimed to provide a theoretical basis for postmenopausal women seeking medical treatment or receiving preventive health care for rabies.

## MATERIALS AND METHODS

### Cells and virus strains

BHK-21 cells (baby hamster kidney fibroblasts) and LBNSE virus strains were stored in our laboratory; Neuro-2a cells (mouse neuroma blast cells) were purchased from ATCC. BHK-21 and Neuro-2a cells were cultured in Dulbecco’s modified Eagle’s medium (DMEM; Gibco, Grand Island, NY) supplemented with 10% fetal bovine serum (FBS; Gibco, Grand Island, NY) and 1% penicillin/streptomycin at 37°C with 5% CO_2_.

### Animal ethical statement

Female BALB/c mice aged 8−10 weeks were purchased from Jinan Xingkang Biotechnology Co., Ltd. (Shandong Province, China) and fed in an SPF environment.The procedures used for anesthesia and euthanasia of the study animals followed the tenets of the ARRIVE reporting guidelines (17). The mice were euthanized using CO_2_. After that, samples were collected from euthanized mice.

### Establishment of the ovariectomized (OVX) mouse model

Female BALB/c mice aged 8–10 weeks were randomized into OVX and Sham groups. The mice were anesthetized intraperitoneally with ready-to-use 1.25% tribromoethyl alcohol solution (0.2 mL per 10 g mouse body weight) (Nanjing Aibei Biotechnology Co., Ltd., Jiangsu Province, China). The OVX group underwent bilateral ovariectomy, while fatty tissues of the same size around the ovaries were removed in the Sham group. Two weeks after the operation, blood samples were collected from the mice (*n* = 4 per group), and the estradiol concentration was monitored via 17β-estradiol enzyme-linked immunosorbent assay (ELISA) (Enzo ADI-900-174) following the manufacturer’s instructions.

### Immunization and pathogenicity

**Fig 1:**
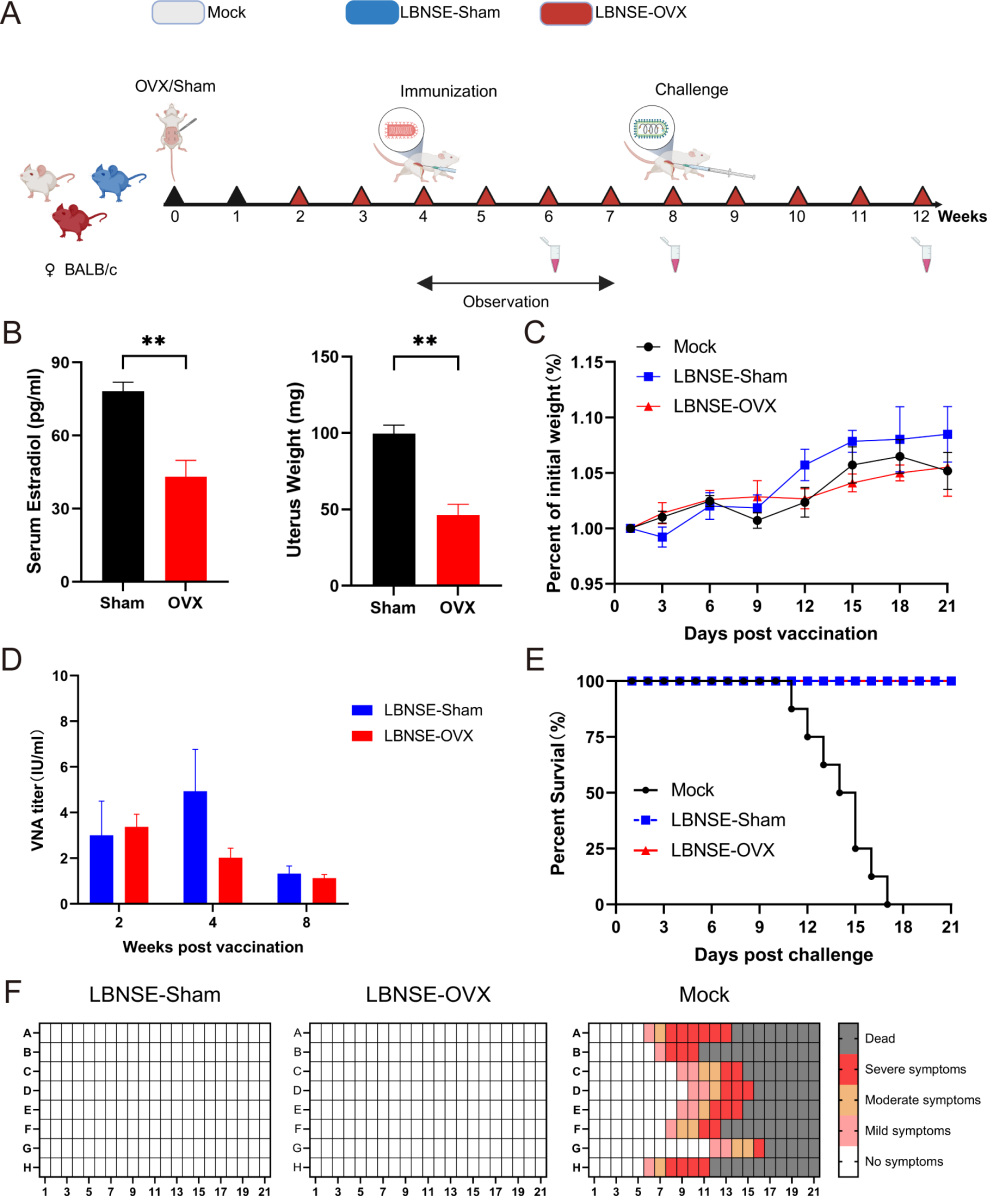
Pathogenicity and immunogenicity of rLBNSE in OVX, Sham and Mock BALB/c mice. (A) Animal experimental design. Female 8–10-week-old BALB/c mice underwent bilateral OVX and Sham surgery separately. Four weeks after surgery, the mice were injected intramuscularly (i.m.) with 100 μl of rLBNSE (equivalent to 1×10^8.5^ TCID_50_ virus) or DMEM (Mock). The status of the immunized mice was monitored continuously for 21 days, and blood samples were collected at 2, 4 and 8 weeks after immunization. The viral challenge was performed at 4 weeks postimmunization. (B) Serum estradiol concentrations and uterus weights of Sham and OVX mice (n=4 in each group) were measured at 2 weeks after surgery. (C) Body weights and clinical signs of the immunized OVX, Sham and Mock mice (n = 8 in each group) were recorded daily for 21 days after immunization. (D) The titers of rabies-specific VNAs in the sera of immunized OVX, Sham and Mock mice were detected (n = 8 in each group at each time point) at 2, 4, and 8 weeks after immunization. The immunized OVX, Sham and Mock mice (n = 8 in each group) were challenged with a 100-fold lethal dose (MLD_50_) of the street RABV HuNPB3 at 4 weeks after rLBNSE immunization and monitored for 21 days. The survival rates (E) and clinical symptoms (F) of immunized OVX, Sham and Mock mice after the challenge were recorded. The data are presented as the means ± SEMs, and significant differences between groups are indicated as **P < 0.01.

Female BALB/c mice (8‒10 weeks old) were randomly divided into three groups (*n* = 8 per group at each time point): Mock, LBNSE-Sham, and LBNSE-OVX. One hundred microliters of virus mixture (equivalent to 1 × 10^8.5^ TCID_50_ rLBNSE virus) or DMEM was injected intramuscularly (i.m.) into the corresponding groups. The immunization protocol is presented in Fig. 1A. The body weights and health status of the mice were recorded to evaluate the pathogenicity of LBNSE in the three groups for 21 days after immunization. Four weeks after immunization, the mice were challenged with a 100-fold lethal dose (MLD_50_) of the street RABV HuNPB3 strain. The dietary status, clinical conditions, and survival rates of the mice in the three groups were closely observed and recorded for 21 days after the challenge.

### Viral neutralizing antibody (VNA) determination

At 2, 4, and 8 weeks after immunization, blood samples were collected from BALB/c mice in the Mock, LBNSE-Sham, and LBNSE-OVX groups (*n* = 8 in each group at each time point), and anti-RABV viral neutralizing antibodies (VNAs) were measured via a fluorescent antibody viral neutralization assay (FVAN) as previously described (18).

### Flow cytometry

On days 3, 6, and 9 after immunization (dpi), the inguinal lymph nodes (LNs) or peripheral blood of BALB/c mice in the Mock, LBNSE-Sham, and LBNSE-OVX groups (*n* = 5 in each group at each time point) were collected. Single-cell suspensions were prepared and stained separately with antibodies (BD Biosciences, Franklin, TN, United States) against DCs in LNs (CD80^+^, MHC I^+^, MHC II^+^, CD86^+^ and CD11c^+^) or B cells in LNs or peripheral blood (CD40^+^ and CD19^+^). A flow cytometer (BD FACS Celesta, USA) was used to perform the flow cytometry procedure and collect signals, and FlowJo V10 software was used to analyze the data.

### Enzyme-linked immune spot assay (ELISpot)

At 4 weeks after immunization, spleens were collected from BALB/c mice (*n* = 5 in each group) in the Mock, LBNSE-Sham, and LBNSE-OVX groups, which were euthanized via CO_2_. Lymphocytes in the spleen were dissociated and stimulated with 2 µg of inactivated purified RABV virions for 16–24 h in a 37°C incubator with 5% CO_2_. The number of lymphocytes that secreted IL-4 or IFN-γ was determined with mouse IL-4/IFN-γ ELISpot kits (Dakewe Biotech Co. Ltd., China) following the manufacturer’s instructions. The number of spot-forming cells (SFCs) was calculated after eliminating background responses with an ELISpot plate reader (AID ELISpot reader-iSpot, AID GmbH, Germany).

### Intracellular cytokine staining (ICS)

At 4 weeks after immunization, the immunized Mock, LBNSE-Sham, and LBNSE-OVX mice (*n* = 4 in each group at each time point) were euthanized with CO_2_. The spleens of the three groups were collected, ground with a 70 µm cell strainer, and lysed with 2 × RBC lysis buffer (Solarbio Science & Technology, Beijing, China) to obtain single-cell suspensions. In six-well plates, 2 × 10^6^ cells per well were placed in 1640 medium supplemented with 10% FBS. After stimulation with RABV G protein (10 µg/mL) and a protein transport inhibitor for 8 h at 37°C, the cells were collected and incubated with anti-mouse CD16/CD32 antibodies for 15 min. Then, the cells were stained with anti-CD4 and anti-CD8 monoclonal antibodies, permeabilized, and stained with anti-IFN-γ and anti-IL-4 monoclonal antibodies, respectively, for 30 min at 4°C. A BD FACS Celesta flow cytometer and FlowJo V10 software were used to detect and analyze the staining results.

### Enzyme-linked immunosorbent assay (ELISA)

The levels of IgG1 and IgG2a antibodies against RABV in the mouse serum of the Mock, LBNSE-Sham, and LBNSE-OVX groups (*n* = 5 in each group at each time point) were measured by ELISA at 2, 4, and 8 weeks after immunization. A total of 1 µg of purified RABV G protein per well was precoated in 96-well plates for ELISA, ensuring that the levels of IgG1 and IgG2a in the sera of immunized mice specific to RABV could be measured. After washing and blocking, 100 µL of 10-fold serially diluted sera from the LBNSE-Sham and LBNSE-OVX groups were added and incubated overnight at 4°C. The plates were washed and then incubated at room temperature for 3 h with 100 µL of HRP-conjugated goat anti-mouse IgG1 and IgG2a (Southern Biotech, United States) at a dilution of 1:500. ABTS was added, and the plates were incubated at 37°C for 15 min after washing. The absorbance at 405 nm was measured via a microplate reader (Mutishan MK3, Thermo Scientific). Serum samples from the Mock group that were free of RABV infection were used to adjust the cutoff level. Sample titers were considered positive when the OD values were ≥2.1 cutoff values.

### Statistical analysis

The experiments were repeated, and the data were collected at least three times. The data were analyzed via SPSS 26.0 statistical software (SPSS Inc., Chicago, IL, United States) and are presented as the means ± standard deviations. Graphs were drawn with GraphPad Prism 8 statistical software. Statistical differences among the groups were identified through one-way analysis of variance (ANOVA) and *t*-tests. *P* < 0.05 was considered to indicate statistical significance.

## RESULTS

### The pathogenicity and immunogenicity of the LBNSE virus in OVX mice

To validate whether the estrogen deficiency mouse model was successfully constructed, serum estrogen levels and uterine weight were detected at 2 weeks after surgery. The experimental results are shown in Fig. 1B and C. Compared with those of the SHAM group, the serum estrogen levels and uterine weights of the OVX group were significantly lower, indicating that the estrogen deficiency mouse model was successfully established.

The mice in the Mock, LBNSE-Sham, and LBNSE-OVX groups were observed continuously for 21 days after immunization (Fig. 1C). The physical activity and dietary status of the mice were normal, with no signs of rabies. The body weights of the LBNSE-OVX mice showed an increasing trend similar to that of the Mock and LBNSE-Sham groups, with no significant difference. The results indicated that OVX and LBNSE immunization had no obvious pathological effects on the immunized mice.

The average viral neutralizing antibody (VNA) concentrations of the mice in the LBNSE-Sham group at 2, 4, and 8 weeks were 3, 4.93, and 1.29 IU/mL, respectively. The average VNA concentrations of the LBNSE-OVX mice at 2, 4, and 8 weeks were 3.37, 2.01, and 1.126 IU/mL, respectively. At all three time points, the concentrations in both groups were greater than 0.5 IU/mL, which is the minimum protection value of RABV recommended by the WHO (Fig. 1D). Compared with those in the LBNSE-Sham group, the number of antibodies in the LBNSE-OVX group was slightly lower at 4 weeks, but the difference was not statistically significant (*P* > 0.05). However, at 2 weeks, the VNA concentrations of the LBNSE-OVX mice were similar to those of the LBNSE-Sham mice. The VNA levels in the LBNSE-OVX mice decreased 4 weeks after immunization, and the reduction rate was greater than that in the LBNSE-Sham mice. These data indicate that estrogen deficiency after OVX might lead to a decrease in RABV-neutralizing antibodies within the effective period and that additional measurements might be needed to maintain sufficient VNA production to combat rabies attacks.

To investigate whether ovariectomy affects the infection protection rate of mice, a challenge experiment was conducted 4 weeks after LBNSE immunization, the clinical status of the mice was scored, and the death status of the mice was recorded continuously for 21 days (Fig. 1E and F). All the mice in the LBNSE-Sham and LBNSE-OVX groups survived during the observation period after a lethal challenge with the RABV HuNPB3 strain. The mice in the LBNSE-Sham and LBNSE-OVX groups did not show any clinical manifestations of rabies, whereas the mice in the Mock group presented typical clinical symptoms of rabies, such as closed eyes, paralysis of the hind limbs, a reduced diet, hunched back, and listlessness, and all died within 21 days. The results indicated that LBNSE immunization after OVX completely protected the Sham mice from lethal RABV challenge, similar to LBNSE immunization.

### OVX altered the recruitment and activation of DCs and B cells in mice

**Fig 2:**
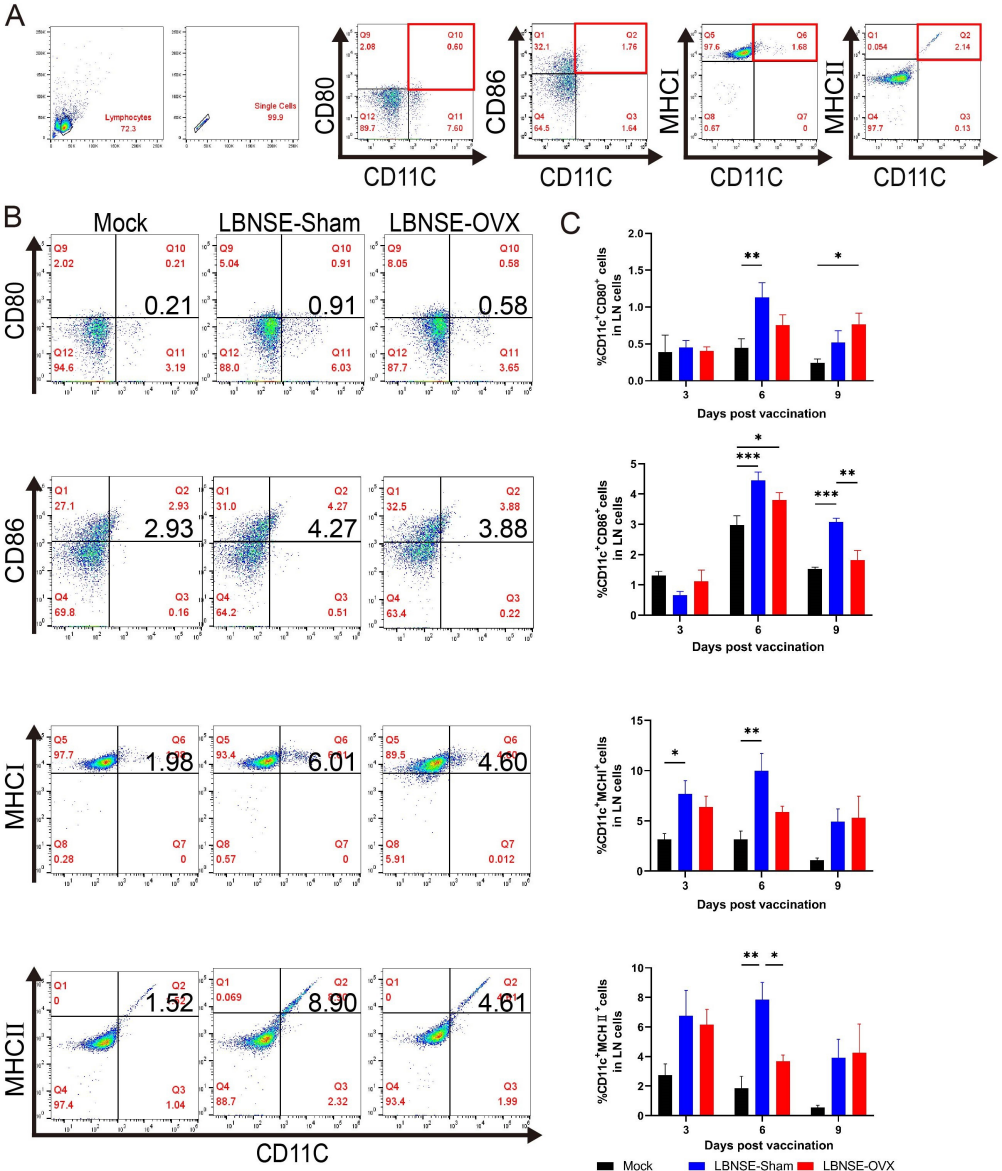
Activation of DCs in the lymph nodes (LNs) of immunized OVX, Sham, and Mock mice. OVX, Sham, and Mock mice (*n* = 5 in each group at each time point) were immunized i.m. with 1 × 10^8.5^ TCID_50_ rabies virus or DMEM in 100 µL. The inguinal lymph nodes (LNs) were collected at 3, 6, and 9 days postimmunization (dpi). Single-cell suspensions were prepared and stained with CD11c^+^, CD80^+^, CD86^+^, MHC-I,^+^ and MHC-II^+^ antibodies against DC activation markers and then analyzed via flow cytometry. (A) Gating strategies for analyzing DCs. (B) Representative flow cytometric data for activated DC analysis. (C) Statistical analysis of the percentages of activated DCs (CD11c^+^CD80^+^, CD11c^+^CD86^+^, CD11c^+^MHC-I^+^, and CD11c^+^MHC-II^+^). The data are presented as the means ± SEMs, and significant differences between groups are indicated as *P < 0.05, **P < 0.01, and ***P < 0.001.

At 3, 6, and 9 days after immunization (dpi), the inguinal lymph nodes (LNs) and peripheral blood of Mock, LBNSE-Sham, and LBNSE-OVX BALB/c mice (*n* = 5 in each group at each time point) were collected and prepared for DC and B-cell flow cytometry detection. The gating strategy (Fig. 2A) and representative flow cytometric plots (Fig. 2B) of CD11c^+^CD80^+^, CD11c^+^CD86^+^, CD11c^+^MHC-I^+^, and CD11c^+^MHC-II^+^ DCs in LNs are presented. As shown in Fig. 2C, the percentages of CD11c^+^CD80^+^ DCs in the LBNSE-Sham group were greater than those in the Mock group at 6 dpi (*P* < 0.01) and LBNSE-OVX at 9 dpi (*P* < 0.05). The percentage of CD11c^+^CD86^+^ DCs in the lymph nodes of the LBNSE-OVX group was significantly lower than that in the lymph nodes of the LBNSE-Sham group at 9 dpi (*P* < 0.01). There were significantly fewer CD11c^+^ MHC-II+ DCs in the lymph nodes of the LBNSE-OVX group than in those of the LBNSE-Sham group at 6 dpi (*P* < 0.05). These data suggested that estrogen deficiency partly inhibited the recruitment/activation of DCs.

**Fig 3:**
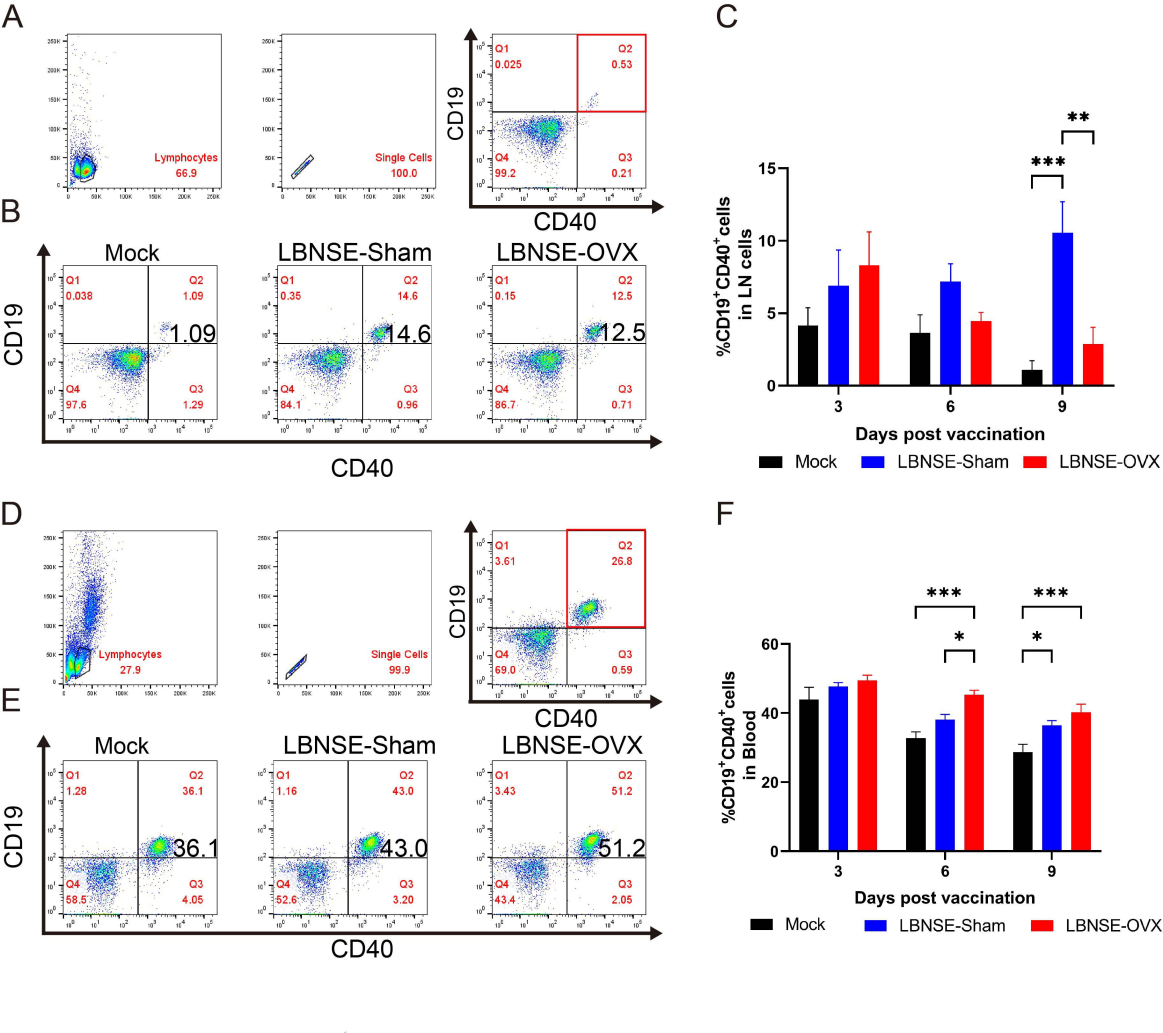
Activations of B cells in inguinal lymph nodes (LNs) and peripheral blood from the immunized Sham, OVX and Mock mice. Sham, OVX and mock mice (n = 5 in each group at each time point) were immunized i.m. with 1×10^8.5^ TCID_50_ rLBNSE virus or DMEM in 100 μl, and the LNs and peripheral blood of immunized sham, OVX and mock mice were collected at 3, 6 and 9 dpi. Single-cell suspensions were prepared and stained with antibodies against B-cell activation markers (CD19 and CD40) and then analyzed by flow cytometry. Gating strategies for B cells from LNs (A) or peripheral blood (D). Representative flow cytometric data for activated B-cell analysis (B, E). Statistical analysis of activated B-cell percentages (CD19^+^CD40^+^ cells) (C, F). The data are presented as the means ± SEMs, and significant differences between groups are indicated as *P < 0.05, **P < 0.01 and ***P < 0.001.

The gating strategies used for B-cell analysis in LNs and peripheral blood are shown in Fig. 3A and D, and representative flow cytometric plots of CD19^+^CD40^+^ B cells are shown in Fig. 3B and E. The percentage of CD19^+^CD40^+^ B cells in LNs (Fig. 3C) in the LBNSE-OVX group was lower than that in the LBNSE-Sham group at 9 dpi (*P* < 0.01), whereas the percentage of CD19^+^CD40^+^ B cells in blood samples (Fig. 3F) in the LBNSE-OVX group was greater than that in the LBNSE-Sham group at 6 dpi (*P* < 0.05). These data revealed that estrogen deficiency may have opposite effects on CD19^+^CD40^+^ B-cell production in the LNs and blood.

### Estrogen deficiency did not suppress specific T-cell responses in mice

**Fig 4:**
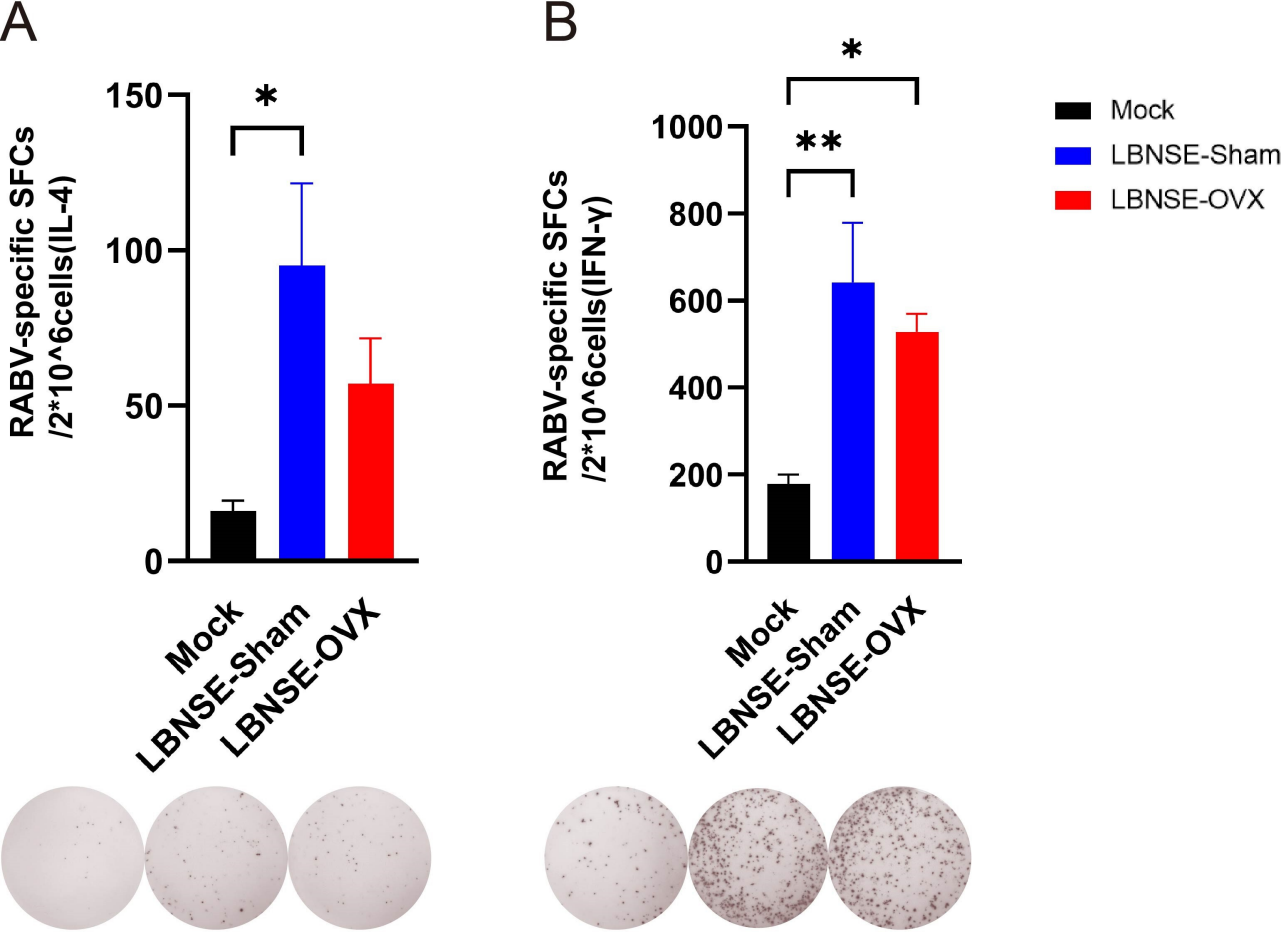
Detection of the production of antigen-specific T cells in the spleens of immunized Sham, OVX and Mock mice via ELISpot. Sham, OVX and Mock mice (n = 5 in each group at each time point) were immunized i.m. with 1×10^8.5^ TCID_50_ rLBNSE or DMEM in 100 μl, and the splenocytes were collected at 4 weeks postimmunization. The inactivated purified RABV virion-specific IL-4^+^ spot-forming cells (SFCs) (A) and IFN-γ^+^ SFCs (B) were detected via ELISpot kits. Representative images of IL-4^+^ or IFN-γ^+^ SFCs are presented below each bar chart. The background response was eliminated when the number of SFCs was calculated. The data are presented as the means ± SEMs, and significant differences between groups are indicated as *P < 0.05 and **P < 0.01.

To investigate the effects of estrogen deficiency on the T-cell immune response induced by LBNSE immunization in Mock, LBNSE-Sham, and LBNSE-OVX BALB/c mice, the spleens of the mice were harvested at 4 weeks after immunization, and the numbers of T cells that secreted IL-4 (Fig. 4A) or IFN-γ (Fig. 4B) against RABV virions were determined via enzyme-linked immunospot (ELISpot) assays. Representative images of IL-4-secreting T cells and IFN-γ-secreting T cells are presented below the bar charts in Fig. 4. The number of IL-4-producing T cells in the LBNSE-Sham group was significantly greater than that in the Mock group (*P* < 0.05). The number of IFN-γ-producing T cells in the LBNSE-Sham (*P* < 0.01) and LBNSE-OVX (*P* < 0.05) groups was significantly greater than that in the Mock group. No significant differences in IL-4-producing or IFN-γ-secreting T cells were found between LBNSE-Sham and LBNSE-OVX mice (*P* > 0.05).

**Fig 5:**
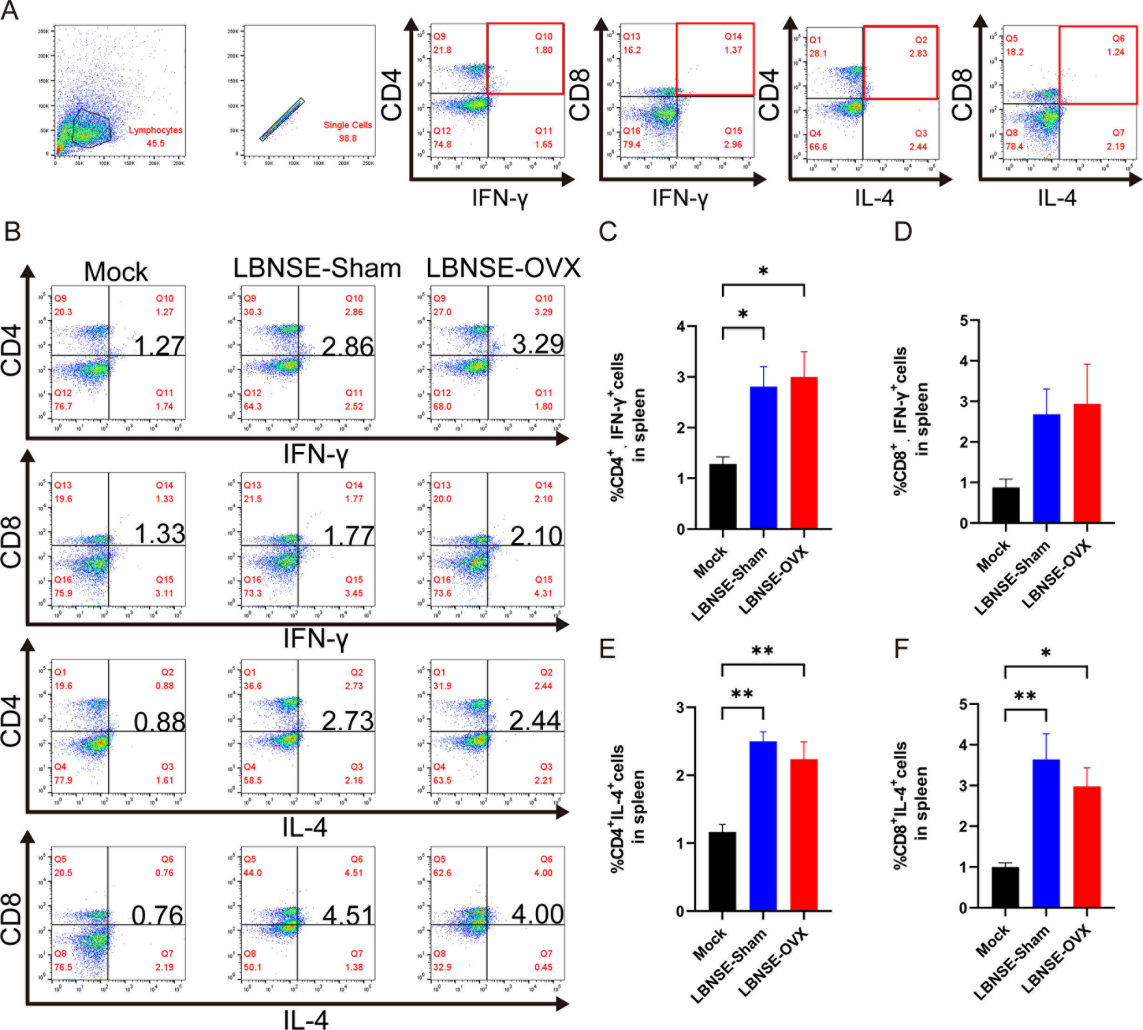
Intracellular cytokine staining (ICS) analysis of antigen-specific CD4^+^ and CD8^+^ T cells in splenocytes from the immunized Sham, OVX, and Mock mice. Sham, OVX, and Mock mice (*n* = 4 in each group at each time point) were immunized intramuscularly (i.m.) with 1 × 10^8.5^ TCID_50_ rLBNSE or DMEM in 100 µL, and the splenocytes were collected at 4 weeks postimmunization. Single-cell suspensions were prepared, and antigen-specific T cells (CD4^+^/CD8^+^; IFN-γ^+^/IL-4^+^) were tested via an ICS assay. (A) Gating strategies for specific T-cell analysis. (B) Representative flow cytometric data for T-cell measurement. (C) Statistical analysis of activated T cells (CD4^+^/CD8^+^; IFN-γ^+^/IL-4^+^). The data are presented as the means ± SEMs, and significant differences between groups are indicated as *P < 0.05 and **P < 0.01.

To further compare specific T-cell activation in the Mock, LBNSE-Sham, and LBNSE-OVX groups at the population level, splenocytes were collected for ICS analysis after 4 weeks of immunization. The flow cytometry results are shown in Fig. 5. The gating strategies used for specific T-cell analysis in the spleen are shown in Fig. 5A, and representative flow cytometric plots of CD4 IFN-γ^+^, CD8^+^ IFN-γ^+^, CD4 IL-4^+^, or CD8^+^ IL-4^+^ T cells are shown in Fig. 5B. As shown in Fig. 5C, the percentages of CD4^+^IFN-γ^+^ T cells, CD4^+^ IL-4^+^ T cells, and CD8 +IL-4+ T cells in the LBNSE-Sham and LBNSE-OVX groups were significantly greater than those in the Mock group (*P* < 0.05). No significant differences in the percentages of CD4^+^IFN-γ^+^, CD8^+^ IFN-γ^+^, CD4^+^IL-4^+^, or CD8^+^ IL-4^+^ T cells were found between the LBNSE-Sham and LBNSE-OVX groups (*P* > 0.05). These results indicated that LBNSE immunization promoted the generation of CD8^+^ T cells and CD4^+^ Th1 and Th2 cells in the spleen of both LBNSE-Sham and LBNSE-OVX mice, but estrogen deficiency did not significantly depress specific T-cell activation in LBNSE-immunized mice.

### Estrogen deficiency-induced specific antibody isotypes in LBNSE-immunized mice

**Fig 6:**
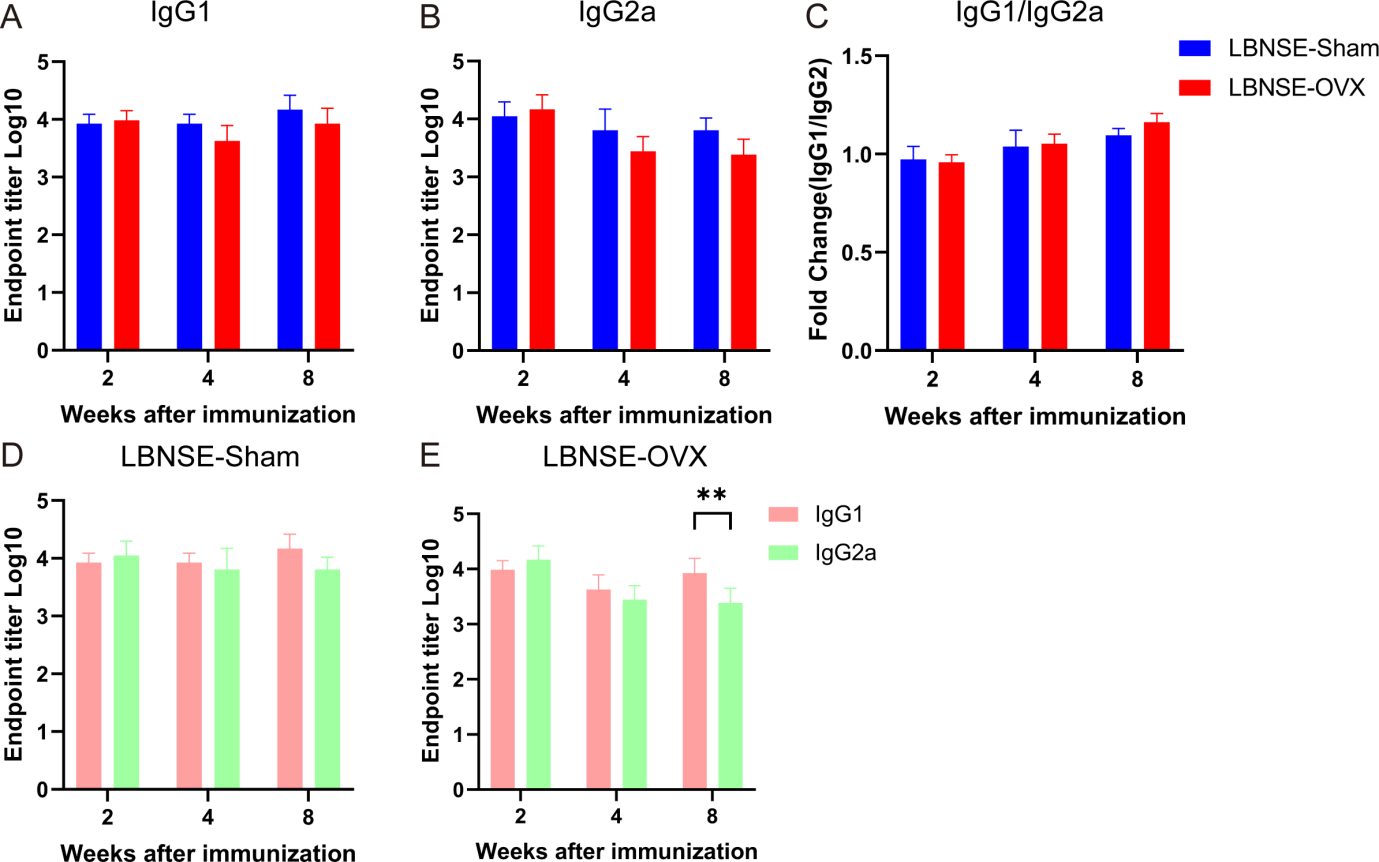
RABV-specific IgG1 and IgG2a subtype detection. Sham, OVX, and Mock mice were immunized i.m. with1 × 10^8.5^ TCID_50_ rLBNSE or DMEM in 100 µL, and blood samples (*n* = 5 in each group at each time point) were collected at 2, 4, and 8 weeks after immunization to detect IgG1 and IgG2a antibodies via ELISA. The titers of RABV-specific IgG1 (A), IgG2a (B), and IgG2a/IgG1 (C) from LBNSE-Sham (D) or LBNSE-OVX (E) immunized mice were statistically analyzed. The data are presented as the means ± SEMs, and significant differences between groups are indicated as **P < 0.01.

Immunoglobulin subtypes are indicators of the potential tendency for Th cell-mediated immune responses (19), and the IFN-γ secreted by Th1 cells induces IgG2a production, whereas the IL-4 secreted by Th2 cells induces IgG1 production. ELISA was used to determine RABV-specific IgG1 and IgG2a antibody subtypes in the serum of mice at 2, 4, and 8 weeks after immunization. As shown in Fig. 6, no significant difference in IgG1 or IgG2a was found between the LBNSE and LBNSE-OVX groups of mice at 2, 4, and 8 weeks after immunization, indicating that the production of RABV-specific IgG1 and IgG2a in OVX mice did not change significantly after LBNSE immunization. LBNSE-OVX can induce Th1- and Th2-type immune responses in mice. By comparing the titers of IgG1 and IgG2a at 2, 4, and 8 weeks after LBNSE immunization, we found that the LBNSE-Sham group mice exhibited even more Th1- and Th2-type immune responses, whereas the LBNSE-OVX mice tended to exhibit Th2-dominant humoral immune responses in the later stage of immunity.

## DISCUSSION

In addition to age, immune changes in menopausal women are closely associated with estrogen deficiency (20). Whether immune alterations induced by estrogen deficiency affect the efficacy of rabies vaccination remains unclear. Compared with inactivated human rabies vaccines, a single dose of modified live RABV vaccine is more immunogenic and can elicit the potent inflammatory response required for effective innate and adaptive immune response induction (21). In this study, we established an OVX mouse model to simulate estrogen deprivation conditions in postmenopausal women and tested the related immune changes after immunization with the modified live RABV vaccine LNBSE. Our results indicate that estrogen deficiency slightly weakens the immune response to rabies immunization but can still provide sufficient effective protection against rabies challenges within the observation period. The viral neutralizing antibody titers in the LBNSE-OVX mice decreased faster than those in the LBNSE-Sham mice did, and the recruitment/activation of DCs and B cells in the lymph nodes and peripheral blood and Th-biased immune responses were altered. Other measurements might be needed to prolong and strengthen the protective effects in postmenopausal women receiving rabies vaccination.

After menopause, the levels of pro-inflammatory cytokines, including interleukin 1β (IL-1β), IL-6, and tissue necrosis factor α (TNF-α), increase, whereas those of CD4^+^ T and B lymphocytes and the cytotoxic activity of NK cells decrease, leading to the mitigation of immune responses to foreign antigens and a predisposition to infection and chronic diseases. Menopause increases COVID-19 severity, invasive mechanical ventilation (IMV) requirements, and disease mortality, especially significantly among Chinese women. The risk of severe COVID-19 is reduced in postmenopausal women receiving hormone replacement therapy (HRT) (22). It has been reported that immune alterations attributed to estrogen deprivation might attenuate vaccine-induced immunity. The ovariectomy of both adult and aged female rhesus macaques reduced T-cell cytokine and IgG production following modified vaccinia Ankara vaccination compared with that in ovary-intact age-matched controls (23). The ability of Herpes simplex virus type 2 vaccination alone to reduce the incidence of genital herpes infection in ovariectomized mice was not as significant as that in naïve control mice, and estradiol complement significantly alleviated the severity of the disease (24). Compared with intact female mice, gonadectomized (gdx) female mice tended to have lower IgG and neutralizing antibody titers for inactivated A/Cal/09 H1N1 vaccine immunization, which could be compensated for by exogenous estradiol supplementation (10). However, universal immune theories cannot be derived because of differences in animal models, immunization times, virus types, and vaccines used in previous studies. The precise effect of estrogen deficiency on rabies vaccination needs to be further studied.

The effects of rabies vaccination rely on prompt antibody-mediated neutralization, and the recommended protective levels of rabies viral neutralizing antibodies (VNAs) are greater than 0.5 IU/mL (25). In this study, the VNA titers of the LNBSE-OVX mice were slightly lower than those of the LNBSE-Sham mice, but the VNA titers in both groups at 2, 4, and 8 weeks postimmunization were greater than 0.5 IU/mL, which might have sufficiently protected the mice from rabies within the observation period. Moreover, the decay rate of VNA levels in LBNSE-OVX mice was faster than that in LBNSE-Sham mice, indicating that more measurements might be required to maintain sufficient VNA production to combat rabies attack.

Previous studies demonstrated that reduced serum antibody titers after seasonal influenza vaccination were produced in older women compared with younger individuals (26). Estrogen treatment can ameliorate inactivated influenza vaccine-induced antibody production in aged females (27) and protect adult female mice from influenza virus infection by increasing the antibody response through ERα signaling (28). Moreover, estrogen supplementation could restore antibody production after inactivated influenza virus split vaccination in gonadectomized young adult mice (29). In contrast to the findings for influenza vaccines, our data showed that estrogen deficiency did not significantly lower VNA production after LNBSE immunization.

Dendritic cells (DCs) are the most powerful antigen-presenting cells (APCs), providing a crucial link between innate and adaptive immunity. DCs and progenitor subsets express both ERα and ERβ, and signaling through these receptors modulates DC differentiation and activity (4, 30). In our study, the percentages of CD11c^+^CD86^+^ DCs at 9 dpi and CD11c^+^ MHC-II^+^ DCs at 6 dpi in the lymph nodes of LBNSE-OVX mice were significantly lower than those in the lymph nodes of LBNSE-Sham mice, indicating that estrogen deficiency may partly impair the antigen-presenting ability of DCs. A previous study showed that E2 increased the surface expression of MHC II, CD40, and CD86 on DCs *in vitro*. However, the expression of activation markers, including MHC II, CD40, and CD86, does not vary among DC populations in the spleens of intact, OVX, and OVX female mice with E2 replacement without any foreign antigen stimulation (31). In our research, the proportions of CD11c^+^CD86^+^ DCs and CD11c^+^ MHC-II+ DCs were significantly lower in the LNs of the LBNSE-OVX group than in those of the LBNSE-Sham group. Further study revealed that cDCs expressing high levels of MHC-II molecules were enriched in the presence of E2 via ER-α, indicating that E2 regulates MHC-II expression on cDC populations via cell-intrinsic ER-α signaling (30). These studies could help explain the decreased proportions of CD11c^+^CD86^+^ and CD11c^+^ MHC-II+ DCs after LBNSE immunization in OVX mice compared with those in intact mice.

ER-α and ER-β are expressed in murine B cells, and E2 may exert direct molecular effects on B cells (32) and can transcriptionally regulate cellular activity and function (33). Elevated estradiol levels are a strong predictor of improved B-cell-mediated immunity and protection against viral infection in females (27). After vaccination, activated B cells undergo rapid proliferation and differentiation within secondary lymphoid tissues, including the spleen and lymph nodes (34). In our study, the percentage of CD19^+^CD40^+^ B cells in the blood of the LBNSE-OVX group was significantly greater than that in the LBNSE-Sham group at 6 dpi. A previous study revealed elevated numbers of circulating lymphocytes in OVX female mice (35), which was in accordance with our study. Although there were fewer CD19^+^CD40^+^ B cells in the LNs of the LBNSE-OVX group than in those of the LBNSE-Sham group at 9 dpi, the overall VNAs were not significantly reduced. Estradiol has been reported to play a central role in modulating antigen presentation, enhancing B-cell responses by increasing the survival of autoreactive B cells and the production of pro-inflammatory cytokines, such as IL-1, IL-6, and TNF-α (32, 36). B cells are essential for regulating immune responses and producing antibodies (37). Estrogen deficiency may negatively regulate DCs and B cells in LNs after LBNSE immunization; however, other compensatory mechanisms might affect antigen presentation and antibody production. Thus, overall VNA production was protective against RABV after LNBSE immunization in OVX mice.

Th1-dependent IFN-γ induces the production of IgG2a, whereas the Th2 cytokine IL-4 stimulates the expression of IgG1 in mice. Research has shown that Th1/Th2 polarization is skewed toward Th1 cells in estrogen-deficient mice caused by either ovariectomy or natural aging (38). Our ELISpot data demonstrated that the number of IL-4-producing T cells or IFN-γ-producing T cells produced in LNBSE-OVX mice was not significantly different from that in LBNSE-Sham mice. Moreover, the ICS results revealed that the proportions of CD4^+^IL-4^+^, CD4^+^IFN-γ^+^, CD8^+^IL-4^+^, and CD8^+^IFN-γ^+^ T cells in LNBSE-OVX mice were not significantly different from those in LNBSE-Sham mice. The detection of immunoglobulins (Igs) of specific isotypes revealed no significant difference in IgG1 or IgG2a between the LBNSE-Sham and LBNSE-OVX groups of mice at 2, 4, and 8 weeks after immunization. LBNSE-OVX mice tended to exhibit Th2-dominant humoral immune responses, whereas the LNBSE-Sham group exhibited more balanced Th1- and Th2-type immune responses. Accumulating evidence has shown that estrogen enhances humoral immunity both in men and women (39) rather than cellular immunity. Our data indicated that humoral immunity was slightly influenced by estrogen deficiency, but cellular immunity did not change significantly at the same time. Considering the limitations of murine models, further safety and protective efficacy evaluations in nonhuman primates or long-term observations are needed.

### Conclusion

In summary, we successfully established an OVX mouse model to simulate estrogen deficiency in menopausal women. LBNSE immunization did not exhibit obvious pathogenicity and had good protective efficacy and immunogenicity against RABV infection in OVX mice, as it did in Sham mice. Estrogen deficiency partly altered the recruitment/activation of DCs and the activation of B cells and did not induce a statistically significant decrease in VNA titers toward the RABV, indicating complete protection. No significant changes were found in the cellular immune response between the LBNSE-OVX and LBNSE-Sham mice. Our study revealed that estrogen deficiency in OVX mice did not significantly affect the humoral immune response after rabies vaccination, which is beneficial for alleviating the anxiety of menopausal women when facing rabies immunization. This study provides a theoretical reference for the immune response of postmenopausal women after receiving rabies vaccinations.
